# Altered composition and function of intestinal microbiota in autism spectrum disorders: a systematic review

**DOI:** 10.1038/s41398-019-0389-6

**Published:** 2019-01-29

**Authors:** Feitong Liu, Jie Li, Fan Wu, Huimin Zheng, Qiongling Peng, Hongwei Zhou

**Affiliations:** 1 State Key Laboratory of Organ Failure Research, Division of Laboratory Medicine, Zhujiang Hospital, Southern Medical University, Guangzhou, 510282 China; 2 Department of Environmental Health, School of Public Health, Southern Medical University, Guangzhou, China; 3 Department of Food and Nutritional Sciences, School of Chemistry, Food and Pharmacy, The University of Reading, Whiteknights, Reading, RG6 6AP UK; 4 Children Healthcare Department, Baoan Maternal and Child Health Hospital, Jinan University, Shenzhen, 518106 China

## Abstract

At present, the pathophysiology of autism spectrum disorder (ASD) remains unclear. Increasing evidence suggested that gut microbiota plays a critical role in gastrointestinal symptoms and behavioral impairment in ASD patients. The primary aim of this systematic review is to investigate potential evidence for the characteristic dysbiosis of gut microbiota in ASD patients compared with healthy controls (HCs). The MEDLINE, EMBASE, Web of Science and Scopus were systematically searched before March 2018. Human studies that compared the composition of gut microbiota in ASD patients and HCs using culture-independent techniques were included. Independent data extraction and quality assessment of studies were conducted according to PRISMA statement and Newcastle-Ottawa Scale. Phylogenetic Investigation of Communities by Reconstruction of Unobserved States (PICRUSt) was used to infer biological functional changes of the shifted microbiota with the available data in four studies. Sixteen studies with a total sample size of 381 ASD patients and 283 HCs were included in this systematic review. The quality of the studies was evaluated as medium to high. The overall changing of gut bacterial community in terms of β-diversity was consistently observed in ASD patients compared with HCs. Furthermore, *Bifidobacterium, Blautia, Dialister, Prevotella, Veillonella*, and *Turicibacter* were consistently decreased, while *Lactobacillus, Bacteroides, Desulfovibrio*, and *Clostridium* were increased in patients with ASD relative to HCs in certain studies. This systematic review demonstrated significant alterations of gut microbiota in ASD patients compared with HCs, strengthen the evidence that dysbiosis of gut microbiota may correlate with behavioral abnormality in ASD patients. However, results of inconsistent changing also existed and further big-sampled well-designed studies are needed. Generally, as a potential mediator of risk factors, the gut microbiota could be a novel target for ASD patients in the future.

## Introduction

Autism spectrum disorder (ASD) is a complex, pervasive neurobiological disorder, characterized by impaired social and communication skills, as well as stereotyped behaviors and restricted patterns of interests^1^. ASD includes autism (AD), Asperger’s Syndrome, and Pervasive Development Disorder Not Otherwise Specified (PDD-NOS). According to recent estimate, the prevalence of ASD is elevating with 1–2% of children currently diagnosed worldwide^2^. The etiology of ASD remains unclear and appears to involve a complicated interaction of genetic and environmental factors^3,4^. By estimate, the heritability including de novo mutations, common variants, and short nucleotide polymorphisms identified in ASD cases altogether accounts for approximately 50% of the disorder^5,6^. As well, the possibility for environmental risk factors and related medical comorbidities which contribute to core neurobehavioral symptoms of the disorder has been highlighted by many studies. Among the comorbidities in ASD, gastrointestinal (GI) symptoms are quite common, such as diarrhea, constipation, and commutative diarrhea/constipation, they are also correlated with the severity of the neurobehavioral disorder^7^. The association of ASD with great prevalence of GI symptoms is spurring an intensive search of the ASD gut microbiota. There is growing evidence demonstrating that disturbances in the pathway underlying the microbiota-gut-brain axis, especially the disordered gut microbiota, may result in neurobehavioral and intestinal dysfunction in ASD patients^8,9^. Gut microbiota makes critical contribution to maintaining the integrity of intestinal epithelia, protecting intestinal barrier and preventing bacterial LPS and other toxins into bloodstream. It has been confirmed that systematic inflammation by LPS induced behavioral impairment and damaged the blood-brain barrier in animal models^10,11^. Conversely, gut microbiota reconstitution with probiotics could alter blood metabolic profiles, remediate gut permeability and improve ASD-related behaviors in mice model^12^. Moreover, gut microbiota may regulate the central nervous system (CNS) activities through neural, immune and endocrine pathways. For example, gut microbiota can regulate the hypothalamic-pituitary-adrenal (HPA) axis^13^, and produce many chemicals affecting brain function (e.g., serotonin, dopamine, r-aminobutyric acid, SCFAs, and p-cresol)^14,15^.

ASD individuals vary widely in clinical presentation, severity and treatment response. The complexity is motivating an exploration to identify biological factor helps to achieve earlier diagnoses and predict clinical prognosis. Thus, the gut microbiota in ASD patients has gained growing attention as a potential mediator of risk factors. So far, several case-control studies aiming to observe the aberrant gut microbiota have been performed^16–32^. Moreover, a recent open-label study has indicated that fecal microbiota transfer (FMT) therapy alters gut ecosystem and improves GI and neurobehavioral symptoms in ASD patients^19^. All these results have gained an insight into the potential mechanisms of gut microbiota in ASD. At present, no systematic review has addressed the evidence of the altered gut microbiota in ASD patients focusing on culture-independent methods especially the high-throughput sequencing techniques. These techniques enable the identification of previously unknown bacterial species, thereby provide novel insights into the compositional diversity and the functional capacity of gut microbiota.

The aim of this systematic review is to explore the current evidence for the alteration of gut microbiota in ASD patients compared with HCs using culture-independent techniques.

## Materials and methods

### Protocol

We conducted the systematic review to evaluate the altered gut microbiota in ASD patients compared with HCs. Available literatures were identified and examined as a systematic review but not a meta-analysis due to the heterogeneity of methods and results. To report this systematic review, the method was consistent with the PRISMA statement guidelines (Preferred Reporting Items for Systematic Reviews and Meta-Analysis), and the protocol was registered at PROSPERO (registration number: CRD42017060769).

### Selection criteria

Studies compared gut microbiota in ASD patients and HCs were included. The inclusion criteria were as follows: (1) ASD diagnosis with definite criteria; (2) the age of participants ranged from 2 to 18; (3) detection of gut microbiota with gut biopsy or fecal samples; (4) metagenomic sequencing, 16S rDNA sequencing, quantitative real-time PCR techniques (qPCR) or FISH. The exclusion criteria were as follows: (1) medicated participants; (2) failure to provide data for the microbiota; (3) culture-dependent methods; (4) intervention studies without initial data or reviews; (5) duplicate publications.

### Search strategy and study selection

A systematic search was conducted using MEDLINE, EMBASE, Web of Science and Scopus for the studies published before March 2018. The reference lists of all identified studies that matched the key search terms were manually searched for relevant trials. The specific search strategy was: (Autism OR autistic OR ASD) AND (microbiome OR microbiota OR microflora OR flora). At the beginning of study selection, irrelevant articles were excluded via an assessment of the tittle, abstract and keywords. The full-text of potentially relevant studies was then retrieved. Following the elimination of duplicates, two independent authors (FL and JL) assessed the articles for eligibility considering established criteria detailed above. Any disagreement between authors were resolved by discussion until consensus was achieved.

### Data extraction

Data for gut microbiota in eligible studies were extracted to the Excel spreadsheet. The following information was extracted: author; publication year; country of origin; the characteristics of case and control (including sample size, mean age, sex ratio, GI symptom and the diagnosis criteria for ASD); the method for microbiota analysis (including sample source, DNA extraction, the information of PCR or FISH or sequencing and the referred database); outcomes (including the differences of overall microbiota structure and the specific bacteria). Besides, the raw sequencing data and biom files were also collected in studies using high-throughput sequencing methods. Two authors (FL and JL) independently extracted data from the selected articles, and the data was then cross-checked for accuracy (FW).

### Quality assessment

Studies included in this systematic review were carefully evaluated for the methodological quality and the risk of bias by two authors (FL and JL). Study quality was assessed on the basis of Newcastle-Ottawa Scale (NOS) for case-control studies. NOS included three domains: selection, comparability and exposure criteria. The selection criteria included four aspects: (1) adequate definition of the cases; (2) representativeness of the cases; (3) selection of controls; (4) definition of controls. The comparability criteria included comparability of case and controls according to the design and analysis. The exposure criteria included three aspects: (1) ascertainment of exposure; (2) same method of ascertainment for cases and controls; (3) non-response rate.

### Summary measures and secondary bioinformatics analysis

The overall microbiota structure, including α-diversity, β-diversity (compositional dissimilarity), the relative abundance of the specific genus and the quantity of specific bacteria by q-PCR or FISH were the primary outcomes. Moreover, to explore the potential function of gut microbiota in ASD patients, the Linear Discriminant Analysis (LDA) effect size (LEfSe) was applied to the relative abundance of Kyoto Encyclopedia of Genes and Genomes (KEGG) pathways predicted by using Phylogenetic Investigation of Communities by Reconstruction of Unobserved States (PICRUSt)^33^. As for the study provided with raw sequencing data, we re-clustered the available data in QIIME with the command of pick_closed_reference_otus.py with the Greengenes reference version 13_8. The operational taxonomic unit (OTU) picking method is Usearch61. As for the study provided OTU biome file, we directly used the biome file to analysis.

## Results

### Study selection

**Fig. 1:**
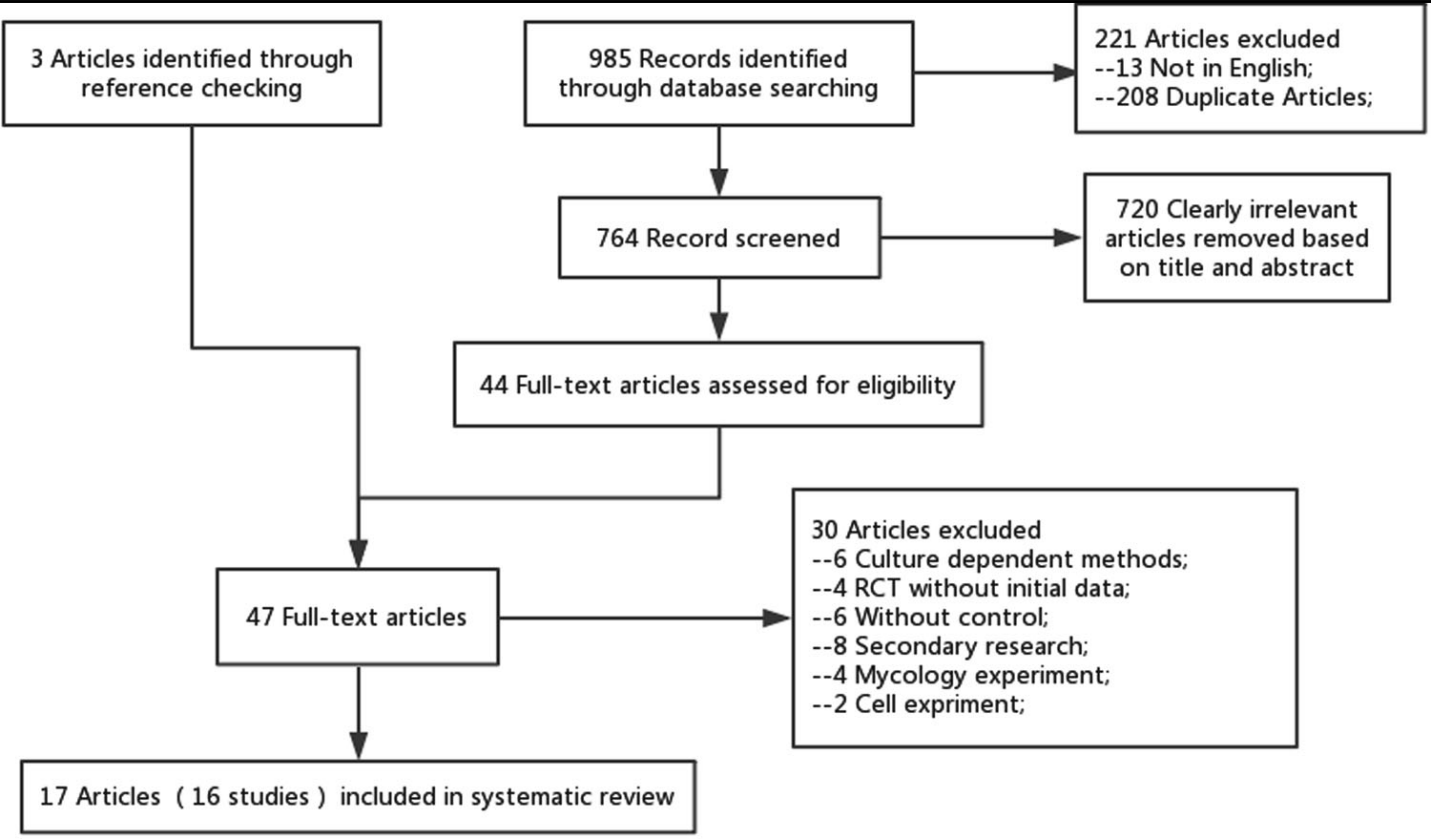
Flow chart of identification, exclusion and inclusion of eligible studies. Flow chart indicates the progression of trials through each stage of the selection process

In total, 985 records were identified through the electronic search. 208 duplicate articles and 13 articles that were not published in English were discarded. 44 full-text articles were retrieved for eligibility following the exclusion of tittles and abstracts that were not relevant to the research. Three additional papers were identified via checking the references of relevant articles. The remaining 47 full-text papers were further assessed according to the fore-mentioned criteria, resulting in the exclusion of 30 papers due to the following reasons: culture-dependent method, randomized controlled trials (RCTs) without initial data, without control group, secondary research; mycology and cell experiment. Following the selection process (Fig. 1), 17 articles remained and were included in the systematic review^16–32^. Among them, William et al.^24,25^ reported one study in two different articles, so did Wang et al.^29,30^. It is also noteworthy that one article^23^ included two group of ASD simultaneously: AD and PDD-NOS, actually counted as two case-control studies. Thus, a total of 17 articles (16 studies) were included.

### Study characteristics

Sixteen studies were published in English journals between 2005 and 2018. Eight studies were conducted in USA^16,17,19,21,22,24,27,31^, three in Italy^18,23^, two in Australia^26,29^, one in UK^32^, one in Japan^20^ and one in Slovakia^28^. The characteristics and main results of studies were outlined in Table 1. A total of 381 ASD patients and 283 HCs were included, with sample size ranged from 12 to 104. Of 283 HCs, 107 subjects were healthy siblings^21,23,26^. The age of ASD patients ranged from 2–18 and the proportion of male ranged from 77.5–100%. Concerning about GI symptoms, five studies^17,19,22,24,27^ reported all of the ASD patients had GI symptoms, seven studies^16,18,21,26,28,29,32^ reported that a part of ASD patients had GI symptoms. And three studies^20,23^ conducted in ASD patients without any GI symptoms. Different diagnostic criteria were used: ADOS, DSM-5, ABD, ADI-R, and CARS. However, two studies^31,32^ did not report the specific diagnostic criteria. As restricted diet is very common in ASD patients, we tried to extract the information of eating habit in ASD and control group. Whereas, only six studies reported dietary information. Strati et al.^18^ reported that all subjects of the study went through a Mediterranean diet. Angelis et al.^23^ reported that the major dietary differences were excluded since each of these pairs of children belonged to the same family unit. The remaining four studies consistently reported that some of the ASD patients had special diets, such as casein-free (CF) or gluten-free (GF) diet^21,22,27,32^. In addition, Son et al.^21^ conducted further nutritional analysis of one-week food diary, no significant difference between two group with respect to daily intake of macronutrients (calories, protein, fats, carbohydrates, sugars or dietary fiber) were noted.

**Table 1:** Characteristics of the trials included

References	Case/Control participants					↔Microbiology assessment			Outcomes (compared with control)
Study country	Sample size	Mean age (Year)	Male ratio (%)	Symptom of GI (%)	Diagnose of ASD	Sample source DNA extraction	PCR or FISH Or Sequencing	Referred Database	Outcomes (compared with control)
Kang^16^	ASD:21	10.1 ± 4.1	15/21	GI symptom were more severe in ASD	ATEC	Fecal sample;	16S rRNA V2-3 region; Genome Sequencer FLX-Titanium System	Greengenes database	↓α diversity;
2018	CON:23	8.4 ± 3.4	22/23		PDD-BI	−80 °C;			β diversity (*P* < 0.05);
USA						PowerSoil® DNA Isolation Kit			Genus:↓Prevotella and Coprococcus
									↓Faecalibacterium, Haemophilus
Luna^17^	ASD:14	Age:4-13	14/14	14/14	ADOS	Rectal biopsy;	16S rRNA V1V3, V4 region; MiSeq Illumina platform	Silva database	β diversity (*P* < 0.05);
2017	CON:15	Age:3-18	12/15	15/15		−80 °C;			↑Clostridiales; Clostridium; Lachnoclostridium; Flavonifractor;
USA						PowerSoil® DNA Isolation Kit			↓Dorea, Blautia, Sutterella;
									Other findings: ↓tryptophan in rectal;
Strati^18^	ASD: 40	11.1 ± 6.8	31/40	5/50	DSM-5	Fecal sample;	V3-V5	Greengenes database	↔ α diversity;β diversity (*P* < 0.05);
2017	CON:40	9.2 ± 7.9	28/40	11/40	ADOS	−80 °C;	454		Phylum:↑Firmicutes/Bacteroidetes ratio;↓Bacteroidetes;
Italy					ABD	FastDNA™ SPIN Kit	pyrosequence		Genus:↑Collinsella, Corynebacterium, Dorea, and Lactobacillus;
									↓Alistipes, Bilophila, Dialister, Parabacteroides and Veillonella;
									Other findings: Candida was more than double in the autistic;
Kang^19^	ASD:18	10.8 ± 1.6	16/18	18/18	ADI-R	Stool samples	16S rRNA V4 region; MiSeq	Greengenes database	↓α diversity;
2017	CON:20	11.4 ± 2.5	18/20	0/20		PowerSoil® DNA Isolation Kit	Illumina platform		Genus:↓Bifidobacterium;
USA									Other findings: ASD had lower fiber consumption;
									ASD were breastfed significantly shorter time;
Inoue^20^	ASD:6	age 3–5	NA	0/6	DSM-5	Fecal sample;	16S rRNA V3-4 region; MiSeq	Greengenes database	Genus:↑Faecalibacterium; ↓Blautia;
2016	CON:6	age 3–5	NA	0/6	PARS	−80 °C;	Illumina platform		Other findings: number of GO biological processes associated with response to viruses were enriched: IFN-γ and type-I IFN signaling pathways.
Japan					M-CHAT	e QuickGene DNA Tissue kit			
Son^21^	ASD:59	10.3 ± 1.8	52/59	25/59	ADOS	Fecal sample;	16S rRNA V1V2, V1V3 region; MiSeq Illumina platform	Silva database	↔α diversity; ↔β diversity;
2015	SIB:44	10.0 ± 1.8 (age 7–14)	21/44	13/44	ADI-R	-80 °C;			↔ the relative abundance of any phylum and genus;
USA						ZR Fecal DNA MiniPre			
Kang^22^	ASD:20	6.7 ± 2.7	18/20	20/20	ADOS	Fecal sample;	16S rRNA V2-3 region; bTEFAP using a 454 FLX Sequencer;	SSURef database	↓α diversity;
2013	CON:20	8.3 ± 4.4(age 3–16)	17/20	0/20	ADI-R	−80 °C; QIAamp			Genus:↓Prevotella, Coprococcus, unclassified Veillonellaceae;
USA					ATEC	DNA Stool Mini Kit			Akkermansia was very high in several autism subjects;
					PDD-BI				
De Angelis^23^	AD:10	age 4–10	NA	0/10	ADI-R	Fecal sample;	16S rRNA V1-3 region; bTEFAP using a 454 FLX Sequencer;	GenBank databases	↑α diversity;β diversity(*P* < 0.05);
2013	SIB:10	age 4–10	NA	0/10	ADOS	−80 °C;			Phylum:↓Firmicutes; Fusobacteria and Verrucomicrobia; Firmicutes/Bacteroidetes ratio;
Italy					CARS	FastDNA Pro Soil-Direct Kit			↑Bacteroidetes;
									Genus: ↓Faecalibacterium, Oscillospira, Bifidobacterium, Fusobacterium, Escherichia, Turicibacter, Eubacterium;
									↑Caloramator, Sarcina, Clostridium, Roseburia, Akkermansia, Shigella, Enterobacter, Dorea;
									Other findings: AD fecal samples contained higher FFA; phenol, 4-(1,1,3,3-tetramethylbutyl)-phenol, p-cresol; SCFAs was lower;
De Angelis^23^	PDD-NOS:10	age 4–10	NA	0/10	ADI-R	Fecal sample;	16S rRNA V1-3 region; bTEFAP using a 454 FLX Sequencer;	GenBank databases	↑α diversity; β diversity(*P* < 0.05);
2013	SIB:10	age 4–10	NA	0/10	ADOS	−80 °C;			Phylum:↓Fusobacteria and Verrucomicrobia;
Italy					CARS	FastDNA Pro Soil-Direct Kit			Genus: ↓Oscillospira, Bacteroides, Fusobacterium, Escherichia, Prevotella, Turicibacter; Clostridium;
									↑Faecalibacterium, Ruminococcus, Roseburia, Alistipes, Dorea;
									Other findings: AD fecal samples contained higher phenol, 4-(1,1,3,3-tetramethylbutyl)-phenol, p-cresol; SCFAs was lower;
William^24,25^	ASD:15	4.5 ± 1.3	15/15	15/15	DSM-5	ileal and cecal biopsies; −80 °C	16S rRNA V2 region; bTEFAP using a 454 FLX Sequencer;	Greengenes database	Phylum:↑Firmicutes/Bacteroidetes ratio; Firmicutes, Betaproteobacteria; ↓Bacteroidetes;
2011,2012	CON:7	4.0 ± 1.1	7/7	7/7	ADI-R		Quantitative Real-time PCR		Family: ↑ Lachnospiraceae and Ruminococcaceae;
USA									Genus:↑Sutterella, Faecalibacterium;Other findings: Presence of Alcaligenaceae in some ASD children but absence in controls.
Gondalia^26^	ASD:51	age 2–12	42/51	28/51	CARS	Stool samples	bTEFAP using a 454 FLX Sequencer;	NA	↔α diversity; ↔β diversity;
2012	SIB:53	age 2–12	19/34	4/53		−20 °C; QIAamp DNA stool kit			↔ the relative abundance of any phylum and genus;
Australia									
Finegold^27^	ASD:11	age 2–13	NA	11/11	CARS	Fecal sample;	bTEFAP using a 454 FLX Sequencer;	RDP-II database	↔ α diversity;β diversity (*P* < 0.05);
2010	CON:8	age 2–13	5/8	NA		-80 °C;QIAamp DNA stool mini kit			Phylum:↑Bacteroidetes, Proteobacteria;
USA									↓Firmicutes, Actinobacteira;
									Genus:↑Alkaliflexus, Desulfovibrio, Acetanaerobacterium,
									Parabacteroides, Bacteroides;
									↓Weissella, Turicibacter, Clostridium, Anaerofilum, Dialister, Pseudoramibacter, Ruminococcus, Streptococcus, Anaerovorax;
									Species:↑ Desulfovibrio spp. and Bacteroides vulgatus;
									↓Bifidobacterium longum, Dialister invisus, Clostridium leptum;
Tomova^28^	ASD:10	age 2–9	9/10	9/10	ICD-10	Stool samples	Quantitative Real-time PCR	NA	↑Lactobacillus;
2015	CON:10	age 2–11	10/10	6/10	CARS	−80 °C; QIAamp DNA stool kit			↑Clostridia cluster l and Desulfovibrio (not significant);
Slovakia					ADI				↓Bacteroidetes/Firmicutes ratio;
Wang^29,30^	ASD:23	10.3 ± 0.8	21/23	9/23	CARS	Fecal sample;	Quantitative Real-time PCR	NA	↑Sutterella spp, Ruminococcus torques (*p* = 0.08);
2011	CON:9	12 ± 1.3	4/9	1/9	DSM-5	−80 °C; A repeat bead beating plus column;			↓Bifidobacterium spp; Akkermansia.muciniphila;
Australia									Other findings: No differences between groups in levels of
									Faecalibacterium prausnitzii;
Song^31^	ASD:15	Age matched	Gender matched	NA	NA	Stool samples	TaqMan	NA	↑Clostridia bolteae, Clostridia and clusters I and XI;
2004	CON:8					−80 °C; QIAamp DNA stool kit	Real-time PCR		
USA									
Helena^32^	ASD:58	7 ± 3.76	48/58	53/58	NA	Fecal sample;	FISH	NA	↑Clostridium histolyticum group (Clostridium clusters I and II);
2005	CON:10	6 ± 2.88	6/10	0/10		−20 °C;			
UK									

The alteration of gut microbiota composition was assessed with fecal samples^16,18–22,25–31^ or gut biopsy^16,23^. Ten studies^16–23,26,27^ assessed gut microbiota by high-throughput molecular approaches: Illumina MiSeq platforms, 454 pyrosequencing or bTEFAP using a 454 FLX Sequencer. Three studies^28–31^ detected gut microbiota with qPCR and one^32^ with FISH approach. One study^24,25^ used both sequencing and qPCR method. Of the studies adopting 16S rDNA-based method, two studies^26,27^ did not report which hypervariable region of the 16S rDNA was targeted and the remaining studies targeted varied sets of regions. Meanwhile, the database used for mapping the sequences were GreenGenes^16,18–20,24^, RDP-2^27^, Silva^17,21^, SSURef^22^ and GenBank^23^, while one study^26^ did not report the database used.

### Risk of bias

16 studies were identified and assessed as medium (6–7) to high (8) quality by the NOS as presented in Table 2. When assessing the quality of selection, the studies of Song et al.^31^ and Helena et al.^32^ lacked an adequate definition of the ASD patients, thus these two studies achieved 3 points in the selection assessment. As for comparability, Luna et al.^17^, Inoue et al.^20^, De Angelis et al.^23^ and William et al.^24,25^ included case and control with better design, such as all participants has consistent GI symptoms at baseline, thus these studies achieved 2 points in the comparability assessment.

**Table 2:** Quality assessment of included studies

First author	Year	Selection	Comparability	Exposure	Total
Kang^16^	2018	4	1	2	7
Luna^17^	2017	4	2	2	8
Strati^18^	2017	4	1	2	7
Kang^19^	2017	4	1	2	7
Inoue^20^	2016	4	2	2	8
Son^21^	2015	4	1	2	7
Kang^22^	2013	4	1	2	7
De Angelis^23^	2013	4	2	2	8
De Angelis^23^	2013	4	2	2	8
William^24,25^	2012	4	2	2	8
Gondalia^26^	2012	4	1	2	7
Finegold^27^	2010	4	1	2	7
Tomova^28^	2015	4	1	2	7
Wang^29,30^	2011	4	1	2	7
Song^31^	2004	3	1	2	6
Helena^32^	2005	3	1	2	6

### Heterogeneity

Methodological sources of heterogeneity included the type of sample (fecal or biopsy), the temperature samples stored, the methods of DNA extraction and the primer used to PCR. Although the included studies had slightly differences in primer selection, there was significant overlap in the key variable and constant regions of the 16S gene. The bacterial identification platform was also a possible source of heterogeneity. 16S rDNA sequencing can quantitatively identify all bacteria present in one sample. However, qPCR and FISH only detected the specific bacteria, which lacked the evaluation of the whole community.

Possible clinical heterogeneity included age, gender, type of control (sibling vs non-sibling) and whether had GI symptoms. Although age structure varied greatly among studies, extensive overlap was found that age of all participants ranged from two to eighteen. In terms of gender, Luna et al.^17^ and William et al.^24^ only included male individuals, the remaining studies included both male and female. Besides, Son et al.^21^, De Angelis et al.^23^ and Gondalia et al.^26^ recruited healthy siblings as control, whereas other studies recruited normal control from the whole population. Considering the impact of GI symptoms on composition of gut microbiota, a potential source of heterogeneity may come from the varied baseline of GI symptoms.

### The altered composition of gut microbiota in ASD patients

In the studies using high-throughput sequencing method, the diversity of species in the samples can be expressed in many ways, including richness, evenness, and α-diversity. α-diversity is the number of species and their proportion within one sampling site. Of the nine studies compared α-diversity, three studies demonstrated that α-diversity has a significant reduction in ASD patients^16,19,22^. In contrast, De Angelis et al.^23^ reported the increased α-diversity was found in both AD and PDD-NOS patients compared with their sibling control. β-diversity means the dissimilarity between communities of two sites or two samples. In this systematic review, ten studies analyzed β-diversity (unweighted UniFrac distance, weighted UniFrac distances, and Bray-Curtis), six of them consistently reported that the microbiota of ASD patients clustered significantly apart from that of HCs^16–18,23,27^.

To assess the specific changing of bacteria in ASD patients, we analyzed the phyla grouping at first. Three studies indicated a clear alteration of the gut bacterial community in ASD patients characterized by a higher *Firmicutes/Bacteroidetes* ratio in ASD than that in HCs due to a significant increase of *Firmicutes* and/or a reduction of *Bacteroidetes*^18,24,28^. Whereas, De Angelis et al.^23^ and Finegold et al.^27^ reported totally opposite results. Phylum of *Fusobacteria* and *Verrucomicrobia* were significantly decreased in patients of ASD (AD and PDD-NOS) in the data of De Angelis et al.^23^ Consistently, William et al.^24^ and Finegold et al.^27^ both reported that the *Proteobacteria* was elevated in ASD patients rather than HCs.

Further analysis of the alterations of genus and species in ASD, four studies consistently demonstrated a significantly decrease of *Bifidobacterium* in patients with ASD relative to HCs^19,23,27,30^. Consistent with this, the abundance of *Blautia*^17,20^, *Dialister*^18,27^, *Prevotella*^16,22,23^, *Turicibacter*^23,27^ and *Veillonella*^18,22^ were all decreased. In contrast, *Lactobacillus*^18,28^, *Bacteroides*^23,27^, and *Desulfovibrio*^27,28^ were all increased in ASD patients rather than controls. Five studies^17,23,28,31,32^ all revealed that there was a significant increase of *Clostridium* in ASD. In addition, De Angelis et al.^23^ indicated that *Oscillopira* decreased and *Roseburia* increased in both AD and PDD-NOS patients. Meanwhile, they also found that some opportunistic pathogen such as *Enterobacter* and *Shigella* were elevated in ASD patients^23^. Whereas, conflicting results were also reported. Two sequencing studies reported that the abundance of *Akkermansia* was elevated in ASD patients^22,23^. Inconsistently, one qPCR study^30^ indicated that lower amount of *Akkermansia muciniphila* was found in feces of ASD patients than HCs. *Dorea*^18,23^ and *Sutterella*^25,29^ were reported increased significantly in ASD patients, but they were reported decreased in another study^17^. *Faecalibacterium*^20,23,24^ and *Ruminococcus*^23,24,29^ were reported increased in ASD patients by three studies, but they were reported reduced in another study^27^.

### The altered function of gut microbiota in ASD patients

**Fig. 2:**
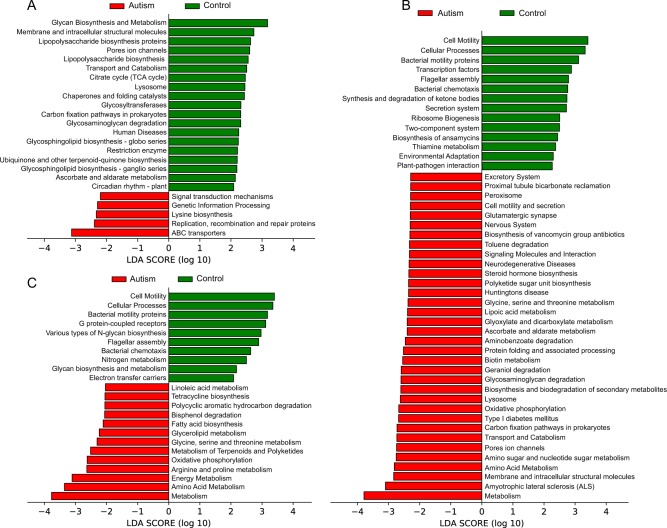
Linear discriminative analysis effect size (LEfSe) of statistically significantKEGG pathways between autism and control in the studies. **a** Strati et al., **b**, **c** two studies of Kang et al. Positive LDA scores (green) are enriched in control while negative LDA scores (red) are enriched in autism

Functional profiles of microbial communities with high-throughput sequencing were predicted by PICRUSt. Raw sequence data was provided in three studies^18,21,22^ and the OTU biome file was provided in one study^19^. Among them, three studies^18,19,22^ indicated the functional modules of gut microbiota in ASD patients were substantially different from that in HCs (Fig. 2). In the study of Strati et al.^18^ (Fig. 2a), the pathways with the highest five discriminative power in HCs were “Glycan Biosynthesis and Metabolism”, “Membrane and intracellular structural molecules”, “Lipopolysaccharide biosynthesis proteins”, “Pores ion channels” and “Lipopolysaccharide biosynthesis”. The pathways with the highest discriminative power in ASD patients were “ABC transporters” under Membrane Transport category, followed by pathways of “Replication, recombination and repair proteins”, “Lysine biosynthesis”, “Genetic Information Processing” and “Signal transduction mechanisms”. In two studies of Kang et al. (Figs. 2b, c)^19,22^, the pathways of “Cell Motility”, “Cellular Processes” and “Bacterial motility proteins” had consistent significant discriminative power in HCs. In addition, “Bacterial chemotaxis” and “Flagellar assembly” were also noted. In ASD patients, the functional modules of metabolism were higher than that in HCs, such as “Oxidative phosphorylation” under Energy Metabolism category and “Glycine, serine and threonine metabolism” under Amino Acid Metabolism. It should be noted that pathways of “Huntingtons disease” and “Amyotrophic lateral sclerosis (ALS)” under Neurodegenerative Diseases category as well as “Glutamatergic synapse” under Nervous System category were also increased in ASD patients. Whereas, in the study of Son et al.^21^, there was no difference of functional modules between ASD patients and HCs, which was accordance with the result of gut microbiota composition.

## Discussion

This systematic review demonstrated that there was consistent evidence for the alterations of gut microbiota in ASD patients compared with HCs. Novel culture-independent techniques that analyzed bacterial DNA offered a unique, more in-depth look into the gut microbiome. Overall, the changed structure of gut bacterial community in terms of β-diversity was observed coherently in ASD patients compared with HCs. Consistently, ASD patients had elevated abundance of *Proteobacteria* rather than HCs. In addition, *Bifidobacterium, Blautia, Dialister, Prevotella, Veillonella*, and *Turicibacter* were consistently decreased, while *Lactobacillus, Bacteroides, Desulfovibrio*, and *Clostridium* were increased in ASD patients relative to HCs.

Several included studies reported the *Firmicutes/Bacteroidetes* ratio due to the alteration of *Firmicutes* and *Bacteroidetes*, but has not reached to any concordant conclusions. Some studies indicated that elevated *Firmicutes /Bacteroidetes* ratio was correlated with inflammatory conditions such as inflammation bowel diseases (IBDs)^34^ and obesity^35^. The elevated *Proteobacteria* phylum was found in ASD patients. It is noted that *Proteobacteria* is a major of Gram-negative bacteria and includes a variety of opportunistic pathogens. Meanwhile, as microbial signature of dysbiosis in gut microbiota, *Proteobacteria* is associated with host inflammation^36^. It is also remarkable that these Gram-negative bacteria produce a potent toxic factor LPS^37^. An animal study indicated that prenatal LPS exposure reduced the level of glutathione in the brain^38^. Glutathione is a significant antioxidant and closely related to detoxification in the brain^39^. Thus, the elevated abundance of *Proteobacteria* in ASD patients need more attention in future studies. As mentioned, the level of some genera (*Bifidobacterium, Blautia, Veillonella* and *Prevotella*) was decreased in ASD patients. Notably, these particular species are known to be versatile carbohydrate metabolizers^40^. *Bifidobacterium* is among the first colonizers of human intestinal and one of the dominant groups in the gut microbiota of breast-fed infants^41^. It can ferment complex polysaccharides to regulate host function and promote health^42,43^, encouraging interest in its use as probiotics. *Blautia* plays an important role in nutrient assimilation^44^ and gut maturation in children^45^. The reduction of these beneficial bacteria in ASD patients may be implicated in the pathogenesis of the disease. On the other hand, overgrowth of *Bacteroides*, *Desulfovibrio*, and *Clostridium* were also found in ASD patients. Indeed, *Bacteroides* is an abundant genus at all ages, from infants to adults^46^. It is the main producer of propionate in the gut, and the abundance of propionate in feces correlates strongly with the abundance of *Bacteroides*^47^. Propionate produced by microbiota is used for gluconeogenesis in liver and represents a source of glucose level for the host^48^. Whereas, a study indicated that neurodevelopmental abnormality in ASD patients accompanied with impaired propionic acid metabolism^49^, which may relate to the changing of propionate-producing bacteria. *Desulfovibrio* produces LPS as well as hydrogen sulfide which could be toxic to intestinal cells under certain circumstance^27,50^. *Clostridium* has been extensively studied in ASD^51,52^ due to its characteristic of producing exotoxins and propionate, which may aggravate the symptoms of ASD^53^. In addition, some species belonging to the *Clostridium* produce p-cresol. This chemical metabolite could cause the reduction of glutathione and reported to be a possible urinary biomarker for autism^54,55^. De Angelis et al.^23^ indicated that *Enterobacter* and *Shigella* were increased in ASD patients, which were positively correlated with the GI symptoms in autism^18^. These opportunistic pathogens have been previously reported to cause or underlie human infections such as bacteremia and intra-abdominal infection^56^. However, the high population of *Lactobacillus* in ASD patients was not expected. *Lactobacillus* has the ability of fermenting a series of carbon sources primarily to lactic acid and is widely recognized as probiotics^57^. Nonetheless, a recent research indicated that *Lactobacillus* was more abundant in T2DM patients than in HCs^58^.

There were still some conflicting results about the alterations of *Akkermansia, Ruminococcus, Sutterella*, and *Faecalibacterium* in ASD patients*. Akkermansia* and *Ruminococcus* are mucin-degrading bacterium and associated with the gut permeability^59,60^. *Sutterella* can regulate mucosal metabolism and intestinal epithelial integrity^25,29^. Changes of mucus-degrading microbes may be related with mucus producing, which could impact the mucosal barrier in the gut. *Faecalibacterium* is regarded as commensal or even beneficial due to its function of producing anti-inflammation butyrate^61^. Thus, the variation of these genera needs to be further explored with the pathogenesis of ASD in future studies.

The predicted biological functions of the observed microbial community in ASD patients were significantly different from that in HCs, but varied among studies. In two accordant studies^19,22^, functional modules of microbiota in HCs mostly involved in cell motility, cellular processes, bacterial motility proteins, bacterial chemotaxis, and flagellar assembly, which indicated the basic physiological maintenance under normal condition. Whereas, in ASD patients, the most enhanced functional module of microbiota was metabolism, including amino acid metabolism, lipid metabolism, carbohydrate metabolism, energy metabolism, cofactors and vitamins metabolism and tetracycline biosynthesis. Functions of xenobiotics degradation such as toluene, aminobenzoate, polycyclic aromatic hydrocarbon, and bisphenol were also enhanced. Moreover, some functional modules involved in neurodegenerative diseases in human, such as Huntington disease and ALS were also raised in ASD patients. So far, a variety of mechanism has been proposed in ASD associated with the function of gut microbiota, including immune activation/dysfunction, bacterial-derived toxin (e.g., LPS, phenols, p-cresol, 4-EPS), metabolites aberrations in fermentation process or products, such as propionic acid (PPA) and other SCFAs, and the dysregulated metabolism of free amino acids^61^. Thus, the functional analysis of gut microbiota may give some hints to these underlying mechanisms and needs to be studied in the future.

### Microbiome reconstitution could be a potential therapy to ASD patients in future

Since gut microbiota appears strongly associated with ASD, the interests in remodeling gut microbiota with diet, antibiotics, prebiotics, probiotics, and FMT are advancing^19,62–65^. In ASD children, an open-label study indicated that treatment with 8 weeks oral vancomycin greatly improved GI symptoms and ASD disorders^64^. A randomized double-blind crossover trial showed that treatment with probiotics resulted in significant differences in the stool consistency compared to placebo and behavior scores compared to baseline^65^. In addition, Buffington et al. reported that maternal high fat diet (MHFD) can induce abnormal social behavior through mediating the dysbiosis of gut microbiota, but reconstituting microbiota with probiotics can correct social deficits in MHFD offspring^66^. Thus, targeting the gut microbiota could be a possible treatment for ASD patients in the future.

### The possible factors for contradictory findings

There are numerous confounding factors that may limit the consensus of the studies analyzed in this systematic review. Geography and dietary habits are main factors that play a great role in microbiome composition. The studies included in this systematic review were conducted in different countries. Besides, implementation of restricted diet is very common in ASD subjects, such as GF and CF diet. Thus, the enormous variation of dietary habit and its effect on the gut microbiota may mask the true picture of the differences. Secondly, the selection of control (that is, sibling vs non-sibling) may also have subtle influence on gut microbiota. Krajmalnik et al. reported that neurotypical (NT) siblings of ASD children have altered microbiome compared to that of unrelated children^67^. It can be an alternative explanation that why studies^21,26^ using NT sibling controls did not find any significant difference, while other studies using non-sibling controls did. Thirdly, the frequent occurrence of GI symptoms in ASD patients may affect the composition of gut microbiota. Hence, the baseline comparability of GI symptoms in case and control, can facilitate a more robust interpretation of the results. Another important consideration is that biogeographic variation of gut microbiota. The samples were collected from different anatomic subsites within the gut tract or feces. Zoetendal et al. reported that using biopsies other than feces allowed to assess the mucosa-epithelia associated microbiota. As biopsies likely established more intimate interplay with the human intestinal epithelium and immune cells^68^. Besides, the different results of gut microbiota may also arise from distinct techniques for DNA isolation, PCR and sequencing, and small-sample sized studies.

### Limitations

Generally, the analyses and results of gut microbiota were relatively difficult to evaluated. It is probably because that the gut microbiota is a new research field that currently depends much on non-parametric statistics and lacks generally accepted standard methods of reporting results^69^. As stated previously, methodological and clinical heterogeneity made it impossible to combine the results of different studies into a meta-analysis. Publication bias is a common challenge within the area of systematic reviews. However, two studies included in the present review provided null findings, showing that the concern about publication bias may be solved in some extent. In addition, only four studies provided the raw sequencing data or biome file for doing PICRUSt analysis, which may result in the loss of bias. Language bias cannot be excluded because our search strategy exclusively based on the English language dominated databases.

## Conclusions

In summary, this systematic review demonstrated there were consistently alterations of gut microbiota in ASD patients compared with HCs, as assessed by culture-independent techniques. It strengthened evidence that dysbiosis of gut microbiota may correlate with behavioral abnormality in ASD. A number of issues resulted in heterogeneity has be drawn, including dietary factors, the selection of controls, frequent occurrence of GI symptoms and sample sources. Thus, more well-designed studies with available sequencing data are needed to better understand the significance of the host interaction with gut microbiota in ASD. Furthermore, as a potential risk factor, the gut microbiome could be a novel target for ASD patients in the future.
